# Acute Viral Hepatitis E Is Associated with the Development of Myocarditis

**DOI:** 10.1155/2015/458056

**Published:** 2015-01-31

**Authors:** M. Premkumar, Devraja Rangegowda, Chitranshu Vashishtha, Vikram Bhatia, Jelen Singh Khumuckham, Badal Kumar

**Affiliations:** ^1^Department of Hepatology, Institute of Liver and Biliary Sciences (ILBS), D-1 Vasant Kunj, New Delhi 110070, India; ^2^Department of Cardiology, Institute of Liver and Biliary Sciences (ILBS), D-1 Vasant Kunj, New Delhi 110070, India

## Abstract

Myocarditis, an inflammatory disease of heart muscle, is an important cause of dilated cardiomyopathy worldwide. Viral infection is an important cause of myocarditis. This condition presents with various symptoms, ranging from minimally symptomatic cases to fatal arrhythmia and cardiogenic shock, and may develop chronic myocarditis and dilated cardiomyopathy in some patients. We report the case of a 26-year-old patient with acute viral hepatitis E who developed symptomatic myocarditis. As far as we could search, this is probably the 3rd case report of this rare association.

## 1. Introduction

Viral hepatitis associated with complications like cholestasis, arthritis, nephropathy, myocarditis, and peripheral neuropathy [1, 2]. Viral myocarditis presents with various symptoms, ranging from minimally symptomatic cases to fatal arrhythmia and cardiogenic shock, and may develop chronic myocarditis and dilated cardiomyopathy in some patients [3, 4]. Hepatitis E is endemic in India, and outbreaks occur following flooding and breakdown of sanitation barriers in monsoon [5, 6]. We report a case of probable myocarditis secondary to hepatitis E. As far as we could search, this is probably the 3rd case report of this rare association [7, 8]. We also report two similar cases with viral hepatitis E with suspected myocarditis.

## 2. Case 1

**Figure 1:**
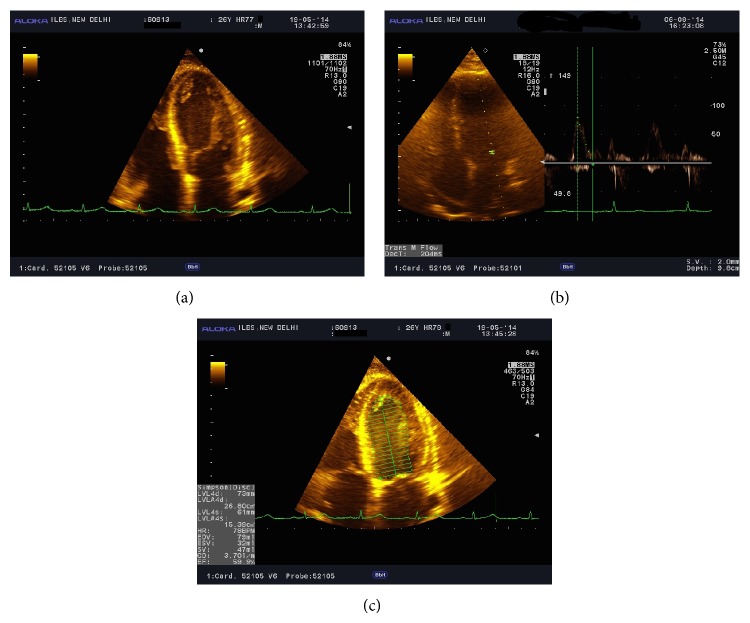
(a) ECHO images showing global hypokinesia in case 1. (b) and (c) ECHO image of patient 1.

**Figure 2:**
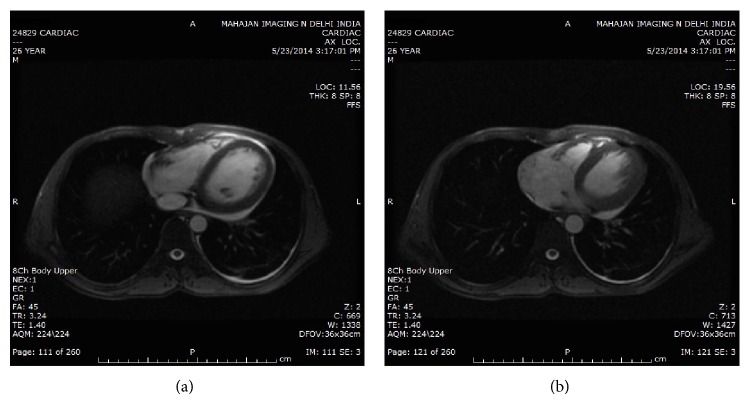
(a) and (b) Cardiac MRI of patient 1.

**Figure 3:**
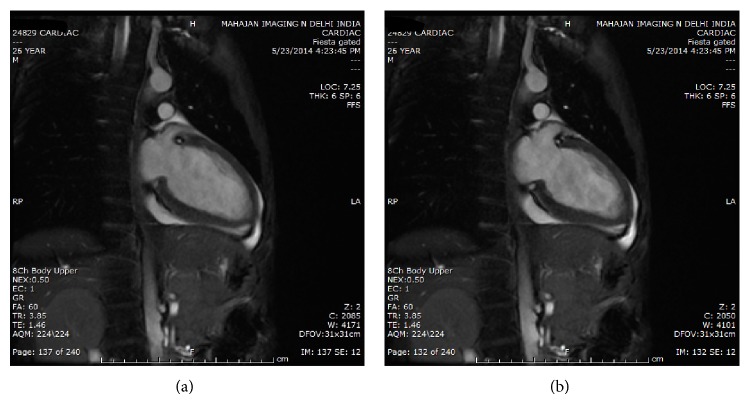
(a) Cardiac MRI of patient. (b) Coronal view cardiac MRI of patient 1.

We report the case of a 26-year-old male who presented to us with the complaint of fever for 7 days which was insidious in onset, low grade without chills, and rigors. He also noted diarrhea for 5 days which was watery, 4-5 times per day associated with diffuse abdominal discomfort but not associated with the passage of blood or mucous. He then noticed jaundice and passage of dark urine for 3 days without any decrease in the frequency or volume of urine. There was no prior history of blood in urine or cola colored urine or burning micturition. There was no history of jaundice, pruritus, clay stools, melaena, hematemesis, abdominal distension, or altered sensorium. He reported only an occasional intake of ethanol. The patient denied intake of indigenous medications or intoxication. The patient did not report any past major surgeries, blood transfusions, or IV drug abuse prior to onset of the disease. He did not report any history of diabetes, hypertension, tuberculosis, thyroid disease, trauma, exposure to industrial toxins or radiation, blood or blood component therapy, bleeding disorders, promiscuity, or similar complaints in the family or neighbourhood. At the time of admission, he was conscious, was oriented, and was febrile. His blood pressure was 110/70 mm of Hg in the right arm, pulse-108/min. He was icteric and did not have pallor, clubbing, cyanosis, pedal edema, or lymphadenopathy. He did not have any skin rash or stigmata of chronic liver disease such as spider angioma, palmar erythema, and parotid enlargement. His initial lab data revealed a hemoglobin of 12 g/dL, total leucocyte counts 11330/dL, and INR (international normalized ratio) of 2.69. His serum bilirubin was 4.5 mg/dL with predominant direct fraction of 3 mg/dL and indirect fraction of 1.5 mg/dL. Liver enzymes showed aspartate transaminase (AST) 452 IU/L, alanine transaminase (ALT) 2750 IU/L, alkaline phosphatase 254, and gamma glutamyl transferase (GGT) 169. Serum albumin was reduced at 2.2 g/dL with globulins 2.7 g/dL. His blood urea was 132 mg/dL with serum creatinine levels of 9.37 mg/dL with normal serum electrolytes suggestive of acute kidney injury. His IgM anti-HEV was positive and serology for hepatitis A, hepatitis B, hepatitis C, HIV, dengue, and* Leptospira* was negative. His peripheral blood smear and rapid malaria test was negative. Due to his deranged renal functions, he was started on slow low efficiency dialysis (SLED) sessions and gradually his urine output improved over the next few days. He was managed conservatively with IV antibiotics, IV fluids, nutritional therapy, and other supportive measures. After two sessions of SLED, he progressed to a polyuric phase, so dialysis was stopped and patient was managed conservatively. On day 6 of presentation, he developed fever again with left sided pleuritic pain and sudden onset of shortness of breath. He was found to be restless and dyspneic and on auscultation he had an S 3 gallop rhythm. Due to increasing respiratory distress, he was intubated and required mechanical ventilation for 3 days. Arterial blood gas analysis revealed type 1 respiratory failure with hypoxemia, and ECG showed sinus tachycardia and dynamic ST-T changes. Chest X-ray was suggestive of pulmonary congestion. Urgent 2D echocardiography revealed global hypokinesia, with normal LV and RV size, but ejection fraction was reduced to 25–30% with preserved right ventricular function. Troponin I was positive at 0.5 ng/mL and creatinine kinase-MB fraction levels were increased at 68 IU/L, 28% of total CK. This was suggestive of myocarditis. He was followed up by daily echocardiograms (see Figure 1). Over a period of 2 days, he gradually improved and was weaned off mechanical ventilation. His ejection fraction improved to 45% by day 3 and 60% with normal ventricular function by day 6. We performed a cardiac magnetic resonance imaging study on day 10 of myocarditis, but by then there was only marginal hypokinesia of the lateral left ventricular wall (see Figures 2 and 3). He was discharged on day 11 of hospital stay.

## 3. Case 2

The second case is that of a 22-year-old male with acute viral hepatitis E. He presented with a week long history of fever followed by jaundice and decreased urine output. At presentation, his total bilirubin level was 7.1 g/dL enzymes AST 945 U/L and ALT 268 U/L. This patient also had acute kidney injury; urea of 88 mg/dL; and creatinine of 3.1 mg/dL, which normalized on conservative management. He was found to have persistent bradycardia for 3 days and was dyspneic at rest, and cardiac enzymes (CK-MB) were elevated on day 3 of admission, and echocardiography revealed global hypokinesia with a reduced ejection fraction of 50%. However, he rapidly improved over the next two days and subsequent cardiac evaluation was normal by day 10 of his illness. Hence he was screened only by serial echocardiography. Cardiac MRI was not done in this patient due to financial constraints.

## 4. Case 3

The third case is of a 24-year-old male patient with acute liver failure secondary to viral hepatitis E. He presented with fever, jaundice, and rapid progression to encephalopathy. This patient had elevated cardiac enzymes, that is, CK-MB, but his troponin I level was <1 ng/mL. ECG showed sinus tachycardia, nonspecific ST-T changes, and T inversions in the lateral leads. This patient was found to have global hypokinesia and LVEF of just 45%. He succumbed to his illness before further diagnostic evaluation could be done.

## 5. Discussion

Myocarditis, an inflammatory disease of heart muscle, is an important cause of dilated cardiomyopathy worldwide. Viral infection is also an important cause of myocarditis. The clinical spectrum of viral cardiomyopathy can be classified as fulminant, acute, or chronic [9, 10].

The progression of viral myocarditis involves three phases [11]. The first phase is characterized by an innate immune response including interferon gamma, natural killer cells, and nitric oxide. Antigen-presenting cells phagocytize released viral particles and cardiac proteins and migrate out of the heart to regional lymph nodes, causing virus-mediated cell lysis and the cardiomyocyte cell death [12]. Most patients recover, but a subset will progress to a second phase, consisting of a virus specific adaptive immune response. In this response, antibodies to viral proteins and to some cardiac proteins (including cardiac myosin, *β*_1_, or muscarinic receptors) are produced, and CD 8+ T cells proliferate. In the third phase, commonly a few weeks after infection, the necrosed myocardium is replaced by diffuse fibrosis, resulting in progressive ventricular dilatation, resulting in chronic cardiac failure. The Dallas criteria [13] remain the standard for diagnosis, but a new clinicopathological staging system has been proposed.

*Expanded Criteria for Diagnosis of Myocarditis [26]*

 
*Suggestive* of myocarditis: 2 positive categories;
 
*compatible* with myocarditis: 3 positive categories;
 
*high probability* of being myocarditis: all 4 positive categories.
 (Any matching feature in category = positive for category.)

*Category I (Clinical Symptoms)*

1. Clinical heart failure,
2. fever,
3. viral prodrome,
4. fatigue,
5. dyspnea on exertion,
6. chest pain,
7. palpitations,
8. presyncope or syncope.

*Category II (Evidence of Cardiac Structural or Functional Perturbation in the Absence of Regional Coronary Ischemia)*

(1)
Echocardiography evidence:
(a)
regional wall motion abnormalities,
(b)
cardiac dilation,
(c)
regional cardiac hypertrophy;
(2)
troponin release:
(a)
high sensitivity (>0.1 ng/mL);
(3)
positive indium In 111 antimyosin scintigraphy;
(4)
normal coronary angiography or
(5)
absence of reversible ischemia by coronary distribution on perfusion scan.

*Category III (Cardiac Magnetic Resonance Imaging)*

1. Increased myocardial T2 signal on inversion recovery sequence,
2. delayed contrast enhancement after gadolinium-DTPA infusion.

*Category IV (Myocardial Biopsy: Pathologic or Molecular Analysis)*

1. Pathology findings compatible with Dallas criteria,
2. presence of viral genome by polymerase chain reaction or in situ hybridization.

The Dallas criteria have standardized the histopathological definition of myocarditis. Despite the EMB yield being only 10% to 20%, EMB findings remain the gold standard for unequivocally establishing the diagnosis. The largest case series of patients with an unexplained cardiomyopathy used biopsy findings to diagnose 111 of 1230 patients (9%) with myocarditis [14]. Notably, less than 10% of 2233 patients with dilated cardiomyopathy referred to the Myocarditis Treatment Trial had EMBs deemed positive by the Dallas criteria [15]. However, several studies have demonstrated strong clinical, echocardiographic, and laboratory evidence of myocarditis amongst patients with negative biopsies [16, 17].

Serum cardiac biomarkers (creatine kinase [CK], troponin I, and troponin T) are routinely measured when myocarditis is suspected. CK or its isoform (CK-MB) is not generally useful for noninvasive screening because of its low predictive value. Lauer et al. reported that only 28 of 80 patients (35%) with suspected myocarditis had elevated troponin levels. Using a serum troponin T cutoff >0.1 ng/mL, the sensitivity for detecting myocarditis is 53%, specificity is 94%, a positive predictive value is 93%, and a negative predictive value is 56% [18].

In our cases, myocarditis or Takotsubo cardiomyopathy was the main differential diagnosis. The first was a case of viral hepatitis E with acute kidney injury requiring dialysis, who developed symptomatic heart failure with pulmonary edema and evidence of cardiac hypokinesia. Takotsubo cardiomyopathy, induced by stress and excess catecholamines, shows a similar clinical course as myocarditis [19]. However, Takotsubo cardiomyopathy usually affects the apical and midventricular myocardium and does not cause diffuse hypokinesis as in our case. Secondly, the patchy diffuse distribution within the subepicardium on CMR is pathognomonic for myocarditis, whereas Takotsubo cardiomyopathy is generally not associated with late gadolinium enhancement [20]. We did not find changes on CMR suggestive of Takotsubo cardiomyopathy. On the basis of these findings, we diagnosed myocarditis. Therefore a combination of noninvasive imaging techniques may obviate the need for a myocardial biopsy to diagnose myocarditis. The second and third cases are only suspicious for myocarditis as though they meet clinical and echocardiographic criteria; we were unable to perform CMR tests in these cases. Neither were we able to perform endomyocardial biopsy in our patients, due to technical risks; all three had coagulopathy due to hepatitis, including one case with acute liver failure. However they further highlight the association of cardiac abnormalities like myocarditis with hepatitis E.

The evidence accumulated so far suggests that the onset of fulminant type 1 involves an immune reaction to an enterovirus. The viral infection would induce a self-perpetuating cycle of cytokine/chemokine overexpression in pancreatic beta cells, leading to apoptosis and destruction. Myocarditis is also commonly induced by viral infections, including the coxsackie virus B [21]. The viruses replicate in the gut and spleen and then spread to the heart. Their replication in the myocardium causes tissue damage amplified by an autoimmune response, leading to heart failure. Matsumori's study sought to detect HCV genomes in formalin-fixed paraffin sections of autopsied hearts from patients with myocarditis, dilated or hypertrophic cardiomyopathy. Among 106 hearts examined, beta-actin gene was amplified in 61 hearts (57.5%). Among the latter, HCV RNA was detected in 13 hearts (21.3%) and negative strands were detected in 4 hearts (6.6%). HCV RNA was found in 4 hearts (33.3%) with myocarditis, in 3 hearts (11.5%) with dilated cardiomyopathy, and in 6 hearts (26.0%) with hypertrophic cardiomyopathy [22, 23].

Several new diagnostic methods, such as cardiac magnetic resonance imaging, are useful for diagnosing myocarditis. Endomyocardial biopsy may be used for patients with acute dilated cardiomyopathy associated with hemodynamic compromise, those with life-threatening arrhythmia, and those whose condition does not respond to conventional supportive therapy. Important prognostic variables include the degree of left and right ventricular dysfunction, heart block, and specific histopathological forms of myocarditis [11, 12].

Therefore, the concomitant viral hepatitis and myocarditis exhibited by our patient may share a common etiology. We did not perform an endomyocardial biopsy in our patient as he had clinically improved, and CMR showed changes suggestive of myocarditis. However we feel that it is pertinent to report that our patient with clinical acute viral hepatitis E and renal dysfunction also developed myocarditis with acute pulmonary edema. Given the fact that viral hepatitis E is endemic in India, many more cases may have gone undetected because of lack of awareness of this association and also because in many cases hepatitis A and hepatitis E infection remain subclinical. Conversely, since we were unable to document myocarditis by means of a definite endomyocardial biopsy, our diagnosis remains clinical with imaging and biochemical supportive evidence. Nonetheless, with increasing number of case reports of association of viral hepatitis A with myocarditis [24, 25], we feel that hepatitis E should also be listed as a possible viral etiology of myocarditis.
